# Practice level factors associated with enhanced engagement with practice facilitators; findings from the heart health now study

**DOI:** 10.1186/s12913-020-05552-4

**Published:** 2020-07-28

**Authors:** Jacqueline R. Halladay, Bryan J. Weiner, Jung In Kim, Darren A. DeWalt, Stephanie Pierson, Jason Fine, Ann Lefebvre, Monique Mackey, Dawn Bergmire, Crystal Cené, Kamal Henderson, Samuel Cykert

**Affiliations:** 1 Department of Family Medicine, School of Medicine, The University of North Carolina at Chapel Hill, 590 Manning Drive, CB #7595, Chapel Hill, NC 27599-7595 USA; 2 Cecil G. Sheps Center for Health Services Research, The University of North Carolina at Chapel Hill, 725 Martin Luther King Jr. Blvd., CB #7590, Chapel Hill, NC 27599-7590 USA; 3 Department of Global Health, University of Washington, Box 357965, Seattle, WA 98195-7965 USA; 4 Department of Statistics, Eberly College of Science, The Pennsylvania State University, University Park, State College, PA USA; 5 Department of Nutritional Sciences, College of Health and Human Development, The Pennsylvania State University, University Park, State College, PA USA; 6 Division of General Medicine and Clinical Epidemiology, Department of Medicine, The University of North Carolina at Chapel Hill, 5034 Old Clinic Bldg, CB #7110, Chapel Hill, NC 27599-7110 USA; 7 Department of Biostatistics, Gilling’s School of Global Public Health, The University of North Carolina at Chapel Hill, 135 Dauer Drive, 3101 McGavran-Greenberg Hall, CB #7420, Chapel Hill, NC 27599-7420 USA; 8 Department of Family Medicine, South Carolina Area Health Education Center, Medical University of South Carolina, 5 Charleston Center, Suite 263, Charleston, SC 29425 USA; 9 Area L AHEC, 1631 S Wesleyan Blvd, Rocky Mount, NC 27804 USA; 10 Division of Cardiology, Department of Medicine, The University of North Carolina at Chapel Hill, 6th Floor, Burnett-Womack Bldg, 160 Dental Circle, CB #7075, Chapel Hill, NC 27599-7075 USA

## Abstract

**Background:**

Practice facilitation is a promising strategy to enhance care processes and outcomes in primary care settings. It requires that practices and their facilitators engage as teams to drive improvement. In this analysis, we explored the practice and facilitator factors associated with greater team engagement at the mid-point of a 12-month practice facilitation intervention focused on implementing cardiovascular prevention activities in practice. Understanding factors associated with greater engagement with facilitators in practice-based quality improvement can assist practice facilitation programs with planning and resource allocation.

**Methods:**

One hundred thirty-six ambulatory care small to medium sized primary care practices that participated in the EvidenceNow initiative’s NC Cooperative, named Heart Health Now (HHN), fit the eligibility criteria for this analysis. We explored the practice and facilitator factors associated with greater team engagement at the mid-point of a 12-month intervention using a retrospective cohort design that included baseline survey data, monthly practice activity implementation data and information about facilitator’s experience. Generalized linear mixed-effects models (GLMMs) identified variables associated with greater odds of team engagement using an ordinal scale for level of team engagement.

**Results:**

Among our practice cohort, over half were clinician-owned and 27% were Federally Qualified Health Centers. The mean number of clinicians was 4.9 (SD 4.2) and approximately 40% of practices were in Medically Underserved Areas (MUA). GLMMs identified a best fit model. The Model presented as odd ratios and 95% confidence intervals suggests greater odds ratios of higher team engagement with greater practice QI leadership 17.31 (5.24–57.19), [0.00], and practice location in a MUA 7.25 (1.8–29.20), [0.005]. No facilitator characteristics were independently associated with greater engagement.

**Conclusions:**

Our analysis provides information for practice facilitation stakeholders to consider when considering which practices may be more amendable to embracing facilitation services.

## Background

Cardiovascular disease (CVD) is the leading cause of death in the USA and is associated with diminished life quality, staggering healthcare costs, and years of life lost [1]. Yet, it is estimated that over 200,000 such deaths could be prevented annually by implementing efforts to reduce CVD risk factors. At the primary care practice level, efforts to recommend appropriate aspirin use, better manage blood pressure and high cholesterol, and encourage smoking cessation are key to reducing CVD, a strategy referred to the “ABCS” of CVD prevention [2].

Primary CVD prevention is the focus of the Evidence Now Initiative funded by the Agency for Healthcare Research and Quality (AHRQ) where seven regional cooperatives, including Heart Health Now (HHN), the North Carolina cooperative, assisted small-medium-sized primary care practices in implementing quality improvement (QI) activities to enhance CVD prevention strategies.

HHN practices were offered on-site support via practice facilitators who joined with practice teams to adopt and implement CVD prevention using QI methods. Activities included abstracting and using ABCS’s-related clinical quality measures from electronic health records (EHR) to drive change, implementing evidenced based care protocols, and enhancing general QI knowledge and skills.

Practice facilitation is an especially promising approach to guide care redesign in primary care settings [3] and has been associated with improved outcomes for patients with a variety of health conditions, including enhanced diagnosis and treatment of asthma, reduced hospitalizations for asthma [4], improved testing behaviors [5], improved office work flows for caring with patients using opioids [6], and enhanced adherence to condition specific [7, 8] and cancer screening guidelines [9]. In Rogers’ Diffusion of Innovations, practice facilitators are referred to as “change agents” [10]. They empower practices to become their own agents of change which distinguishes it from a traditional consulting model, thus practices improve while they simultaneously build internal capacity for change [11]. They enable others to act, as opposed to telling or persuading them to do so [12] and serve as cross-pollinators of ideas and are key resource providers for the practices they serve [13, 14].

Although the evidence for the impact of practice facilitation on outcomes is building, it is a relatively new strategy and questions remain as to what underlies effective facilitation methods. For instance, investigators note challenges with establishing relationships between practice facilitators and practice staff teams; an issue mainly attributed to the continuous array of competing demands that commandeer practice time and resources [15]. For example, in a trial designed to test the impact of practice facilitation on improving diabetes care, disruptions such as staff turnover, moving to a new practice location, and installing a new EHR delayed the facilitation start time by nearly 2 years [3]. Others describe challenges facilitators face getting into practices, establishing trust, and finding practice leaders who prioritize QI work [16, 17]. Suboptimal engagement with practices during the study time period has been identified as a barrier to 1) fully implementing study activities, 2) a facilitator’s ability learn about and work within a practice’s culture, and 3) a practice’s abilities to leverage all that facilitation can offer [16–18]. These studies and others have led to questions regarding if there are unmeasured baseline characteristics that are barriers to engaging with facilitators [18, 19].

In this analysis, we explore one piece of this puzzle using data from the HHN study, specifically if there are practice characteristics and practice facilitator level variables associated with greater levels of engagement between the facilitators and the practices they served. Understanding which factors may enhance or impair facilitator-practice team engagement may help practice facilitation organizations improve project planning, workforce deployment, and reduce delays with project implementation.

## Methods

Study design and setting: HHN is a stepped wedge cluster randomized trial designed to evaluate practice facilitation services on CVD outcomes. Practice recruitment efforts were from May 2015 to December 2015. Five hundred thirty-eight practices offering primary care services in NC with 10 or fewer providers and had an electronic health record were considered for participation in the larger HHN trial. Our focus was on independent practices, Rural Health Centers, and Federally Qualified Health Centers (FQHC), often located in Medically Underserved Areas (MUA) [20] that lack organizational support for workflow redesign and development of tailored quality reports derived from their EHRs. We allowed 21 practice sites owned by health systems to participate because of geographic separation and limited practice support from their parent organizations. Two hundred ninety-two practices ultimately enrolled, and 47 of these dropped out prior to the start of the practice facilitation intervention leaving 245 practices that were offered facilitation services. To be eligible, practices needed to agree to form a QI team, meet with a practice facilitator monthly, and permit access to their EHR data in order to allow for data abstraction and to use this data to create dashboards and other resources regarding clinical quality performance on appropriate use of aspirin, blood pressure control, cholesterol control and smoking cessation (the ABCS’s) measures. Practices were randomized to 7 different site activation months that established the randomized cohorts. Facilitators were expected to engage with practice staff members monthly for 12 consecutive months, but were available for additional phone, email, web conference, and onsite consultations if needed. Facilitators were trained to assign a monthly score to assess each practice’s progress using implementation measures described below. More details of the HHN study are in the study protocol manuscript [21].

The HHN practice facilitators, employed by the North Carolina Area Health Education Centers (NC AHEC) practice support program, base their work on a set of changes that, if implemented, could enhance CVD care processes and outcomes (i.e. key drivers of implementation). The extent of adoption of these drivers are captured using the Key Driver Implementation Scale (KDIS) developed by the NC AHEC practice support program; a tool rooted in the Chronic Care Model [22, 23] and used in NC AHEC projects and program analyses [24–26]. KDIS scales are ordinal scales, generally with 4 or 5 options, that capture when activities are adopted and how fully they are implemented into standard work [26, 27]. The key drivers are part of a larger framework that provides the logic as to how key activities performed within each driver domain can lead to improved outcomes, thus similar to a logic diagram or protocol, but created specifically to support ambulatory care practices in their change efforts. These 4 key driver domains include: 1) optimal use of clinical information systems, 2) adoption of evidence-based care protocols, 3) regular use and referral for patient self-management support and 4) optimization of the care team. The intervention activities that are tested and implemented in practices are purposefully adaptive to address the needs, skills and resources of each clinic. Additionally, the KDIS captures levels of practice Team Engagement (TE) and QI Leadership as defined below and detailed in Tables 1 and 2 respectively. TE indicates that practice facilitators are included as part of a practice team that collectively works to devise, implement, and evaluate small tests of change. The level of this engagement is captured using a 0–3 score.

**Table 1:** Outcome measure: Team Engagement (KDIS Score)

0 – No activity	No engagement.
1 – Occasional meetings	Team meets infrequently to discuss improvement; no practice-wide understanding of improvement work exists.
2 – Regular meetings	Improvement team communicates regularly (through meetings, huddles, email, memos, etc).
3 – Active engagement	Improvement team plans multiple tests simultaneously and communicates findings.

“Adequate Team Engagement (TE)” with a practice facilitator is defined as a mean team engagement score of ≥ 2 calculated as an average in the 4 to 6-month time interval if at least 2 TE scores were available. Team engagement indicates that practice facilitators are included as members of practice teams

**Table 2:** Additional variable descriptions and definitions (and data source^a^)

Variable/item (survey)	Definition
Leadership [23, 26] (KDIS)	0-3 ordinal scale of level of leadership support for QI collected by practice facilitators on their practices on a monthly basis. 0= no leadership support for improvement work, 1= a leader is involved, but no organized improvement structure exists, 2- leadership approaches improvement work on a project basis or as a task to be done by an individual, 3= leadership recognizes QI work as a part of daily routine/culture/ expected job performance.
Organizational readiness [28] (PMS)	Single question on if a practice is “committed to implementing changes after being prompted to consider if they are ready to use care plans, clinical decision support, use CVD risk calculators, manage patient populations and others. Five item Likert response strongly disagree to strongly agree.
Change Capacity [29] PMS)	Practice capacity for Quality Improvement, 14 question- 5 item Likert scale questions that assess how much QI practices already do in CVD and how much support there is for staff training and providing necessary resources for engaging in practice level QI
CVD Priority (PCS)	Single question asking about how highly CVD prevention is prioritized by practice leadership (1= no priority to 10= Highest priority)
Burnout [30] (PMS).	One question item assessing feelings of burnout within the work environment and asking the respondent to use their own definition of burnout. There are 5 response options ranging from no symptoms of burnout to feeling completely burnout.
Adaptive Reserve [31, 32] (PMS)	14 items assessed using a 5-point Likert scale (strongly disagree to strongly agree) where higher scores indicate successful work relationships that lead to flexibility and resilience within a practice. Survey items include questions of clinical staff regarding how well practice teams function well, if they reflect on their work, are willing to change, if problem solving is done well, if there are open communications to discuss what works, if there are growth opportunities, and others.
Number of practice disruptions [33] (PCS)	Asked practice leader if they have had undergone key distractions in the last 12 months, including implementation of a new EHR, moved to a new location, experience clinician and or other staff turnover, was purchased by or joined another organization, implemented a new billing system and “other” for a total of 7 possible changes.

^a^Data sources: *KDIS* Key Driver Implementation Scale, *PMS* Practice Member Survey, *PCS* Practice Characteristics Survey

We focused our analysis at the approximate mid-point of the 12-month intervention in order to evaluate TE when engagement in active implementation is expected, based upon the long tenure of NC AHEC’s practice support program with other statewide QI initiatives and an expectation that if initial engagement was suboptimal, that changes could be made to optimize our ability to retain practices and capture our main results data.

**Fig. 1:**
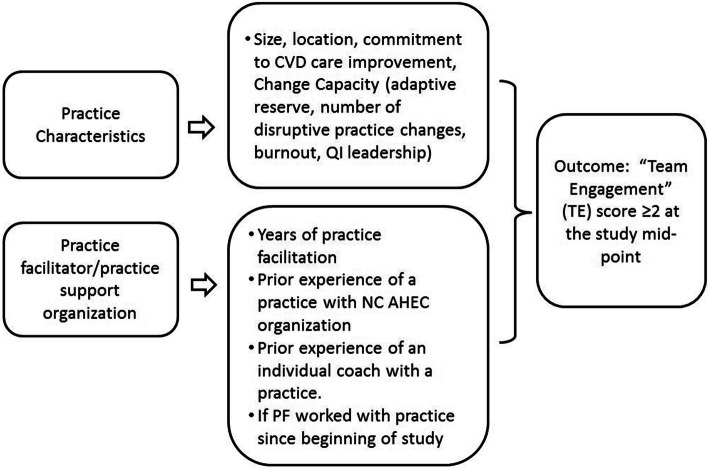
Conceptual Model: HHN Primary Care Practice Engagement with Practice Facilitators and Team Members

To guide our analysis, we developed a conceptual model based upon a White paper by Geonnotti et al. that details strategies for facilitators to enhance their engagement with practices in QI [15]. Our model expands upon this and posits that facilitator and practice-level characteristics are important to creating effective practice staff–practice facilitator project implementation teams. We chose data elements that may enhance or impair the ability of practices to find time and/or have the relevant motivation to engage with facilitators in the HHN trial. These include variables such as practice size, location in a medically underserved area (MUA) or not, payer mix, involvement in other quality initiatives, practice level measures of burnout, readiness and adaptive reserve, leadership support for QI and practice facilitator’s prior experiences and tenure with facilitation (Fig. 1).

### Data sources

**Fig. 2:**
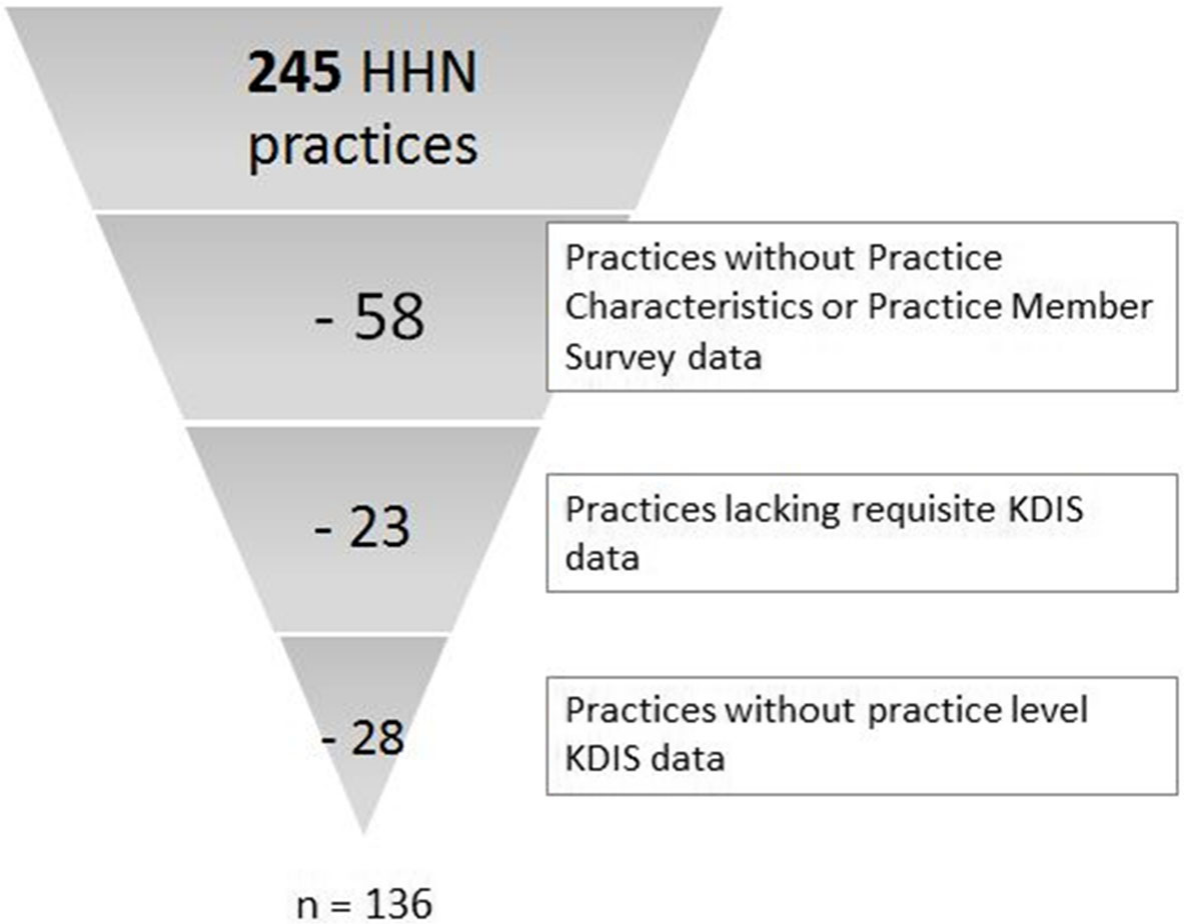
Selection of Analytical Practice Cohort

We collected practice-and facilitator-level data from 4 sources described below and in Table 2. We restricted our analysis to the 136 practices that met our inclusion criteria defined as having 1) responded to both baseline surveys and 2) the requisite practice-level KDIS data (Fig. 2).

#### Baseline practice characteristics survey (PCS)

Completed by a lead practice provider or administrator, this survey captured demographics including number of providers, patient visit volume, payer mix, practice location, number of disruptive changes experienced in the year prior to HHN participation, and experience with other quality initiatives (Table 3).

**Table 3:** Overall Practice Characteristics of 136 HHN Practices and Summary Statistics Comparing Practices with Key Driver Implementation Scale Team Engagement Scores < 2 (*n*= 63) vs. ≥ 2 (*n*=73)

**Practice Characteristics Survey items, [# missing]**	**N or mean (%, SD or range)**^**a**^	**TE < 2** **N or mean (% or SE)**	**TE ≥ 2** **N or mean (% or SE)**	***P*-value** **chi-square or t-test**
Practice Size (# of providers MD, DO, NP PA), [2]	4.9 (4.2)	4.9 (3.4)	4.8 (4.7)	0.99
Practice Ownership Type, [0]				
Clinician-owned Solo or Group Practice	70 (51.5 %)	45 (64.3)	25 (35.7)	
Federally Qualified Health Center (FQHC) or Look-alike/Rural Health Clinic	37 (27.2%)	14 (37.8)	23 (62.2)	
Hospital/Health System/Academic Health Center.	29 (21.3%)	4 (13.8)	25 (86.2)	<.0001
Payer Mix [13], %				
Medicare [13]	30.6% (5-82%)	28.2 (15.1)	32.9 (18.8)	0.13
Medicaid [13]	15.4% (0-50%)	14.9 (10.9)	15.8 (11.1)	0.66
Dual Medicare/Medicaid [14]	9.1% (0-70%)	11.3 (11.9)	7.1 (7.7)	0.02
Commercial [13]	32.5% (0-79%)	35.3 (18.0)	29.8(17.0)	0.09
No insurance [13]	11.8% (0-60%)	9.4 (10.6)	14.1 (15.5)	0.05
Other [14]	1.5% (0-100%)	2.6 (13.4)	0.5 (1.97)	0.22
Patient-Centered Medical Home Recognition, [11]				
Yes	74 (54.4%)	36 (28.8)	38 (30.4)	
No	51 (37.5 %)	25 (20.0)	26 (20.8)	1.00
Patients Seen/Day by Full Time Clinician, [12],	21.3 (10-50)	22.3 (6.4)	20.4 (6.0)	0.09
Practice Location in a Medically Underserved Area (MUA), [0],				
Yes	54 (39.7%)	18 (33.3)	36 (66.7)	
No	82 (60.3%)	45 (54.9)	37 (45.1)	0.02
Change Process Capability Questionnaire (CPCQ) SCORE (scored -28 to 28), [11]	10 (13.3)	9.3 (12.5)	10.5 (14.0)	0.63
CPCQ-Cardiovascular Disease (CVD) Priority (single item, scored 1-10), [1]	7.5 (1.7)	7.4 (1.9)	7.6 (1.6)	0.49
Number of Disruptive Practice Changes (0-7), [0]	1 (1.0)	0.8 (0.9)	1.1 (1.1)	0.06
Prior or Ongoing Involvement in an Accountable Care Organization (ACO), [0]				
Yes	61 (44.9%)	24 (52.0)	37 (48.0)	
No	75 (55.2%)	39 (39.3)	36 (60.7)	0.19
**Key Driver Implementation Scale items**				
^b^KDIS Mean Team Engagement Score of Months 4-6, [0]	1.6 (0.7)	0.98 (0.04)	2.2 (0.04)	<.0001
KDIS Practice Leadership Score (mean of months 4-6 scores, (0-3), [0]	2.0 (0.7)	1.6 (0.6)	2.4 (0.6)	<.0001
**Practice Member Survey Items**				
Adaptive Reserve Score (18 items, aggregate score 0-1), [0]	0.7 (0.1)	0.7 (0.1)	0.7 (0.1)	0.82
Practice Level of Burnout (single item, 0-4), [0]	1.9 (0.6)	1.9 (0.4)	2.0 (0.7)	0.28
Practice Readiness (readiness1) single item, [0]	4.0 (0.5)	4.0 (0.5)	4.0 (0.5)	0.65
**Practice Facilitation Experience Survey Items**				
Years of Experience as a Practice Facilitator, [1]	4.2 (3.7)	4.0 (3.4)	4.3 (4.0)	0.62
Practice with Prior Experience with NCAHEC Practice Support Program, [1]				
Yes	38 (27.9%)	17 (44.7)	21 (55.3)	
No	97 (71.3%)	45 (46.4)	52 (53.6)	1.00
Practice-practice Facilitator Experience Working Together Prior to HHN, [1]				
Yes	9 (6.7%)	4 (44.4)	5 (55.6)	
No	126 (92.7%)	58 (46.0)	68 (54.0)	1.00^^^
Practice facilitator has worked with the practice since the beginning of the project [1]				
Yes	102 (75.0%)	42 (41.2)	60 (58.8)	
No	33 (24.3%)	20 (60.6)	13 (39.4)	0.08

^a^Data provided as absolute numbers or means and standard deviations (SD) for continuous variables and proportions with chi squared test for categorical variables as appropriate. Ranges included for payer mix and number of patients seen per day by a full-time clinician.

^b^outcome measure

^Fisher exact test used where appropriate for small sample sizes, SE=standard error

All values rounded to tenths position; *p*-values rounded to the hundredths unless otherwise stated

#### Baseline practice member survey (PMS)

Practice managers distributed up to 5 surveys to individuals with different roles to obtain a variety of perspectives. These included questions about staff burnout, adaptive reserve and readiness to engage in the study. For our analysis, where more than one person provided responses, practice means were calculated.

#### Practice facilitator data

NC AHEC program leaders provided information about the facilitators such as: 1) years of facilitation experience, 2) existence of prior working relationships between a specific facilitator and a practice, 3) if a practice facilitator originally assigned, remained the practice’s facilitator for the duration of the intervention, and 4) prior working relationship between a practice and the NC AHEC practice support program.

#### Key driver implementation scale (KDIS)

Practice facilitators documented practice KDIS, TE and Leadership scores that reflect observations made during each month. The TE score included 4 options scored from 0 to 3 where a “0” indicated no engagement of the practice team with the facilitator while a “3” indicated that a practice team works with a facilitator in a regular and effective manner (Table 1). Practice Leadership (Table 2) and TE scores were calculated by averaging scores from months 4 to 6.

#### Outcome measure

We define the TE outcome measure as “adequate” if the mean TE score was ≥2 at 6 months; calculated as an average in the 4 to 6-month time interval where at least 2 scores were available. This threshold score was chosen by our NC AHEC leadership and investigator team based upon extensive experience with QI project implementation in both ambulatory practices and health care settings and the score’s representation of a practices’ behavior of having regular QI meetings vs. having irregularly scheduled or no QI meetings (see Table 1. definitions).

#### Analyses

Descriptive statistics summarizing practice characteristics are in Table 3. We estimated the intracluster correlation coefficient (ICC) to measure the clustering effect by comparing the relative levels of between and within facilitator variabilities [34]. The estimated ICC was 0.345, i.e., 34.5% of the total variabilities in TE scores are attributable to differences among facilitators. The generalized linear mixed-effects model (GLMM) with allowing random intercepts per practice facilitator was used in order to adjust for the effect of clustering by practice facilitator. We identified variables associated with TE scores of ≥2 vs. < 2 (Table 4) by fitting all possible models with predictors one-at-a-time. Significant predictors were defined as *p* value equal to or less than 0.1 were included. The final model was selected using the Akaike information criterion (AIC) value with the best fit, thus lowest AIC [35, 36]. The adjusted odds ratio and standard errors of the models are reported in Table 4.

**Table 4:** Univariate and Generalized Linear Mixed Model. Point estimates represent Odds Ratios for HHN practices achieving a mean TE score of ≥ 2 at the study mid-point (~ 6 months)

	GLMM with a single predictor OR (95% CI), [***p*** value]	GLMM best fit Model OR (95% CI), [***p*** value]
For every one-point increase in KDIS leadership score	12.66 (4.75 - 33.77), [0.00]	17.31 (5.24-57.19), [0.00]
For practices located in a Medically Underserved Area (MUA) vs. not in an MUA	5.66 (1.86 – 17.30), [0.002]	7.25 (1.8 – 29.20), [0.005]
For practices that are community health centers/health departments vs. solo/privately owned	6.36 (1.64 – 24.63), [0.007]	n/a
For practices that are Hospital/Health System/Academic Health Center vs. solo/privately owned	5.91 (0.91 – 38.52), [0.063]	n/a
For every 1% increase in percentage of patients with no insurance	1.05 (1.00-1.10), [0.032]	n/a
For every 1% increase in percentage of patients with dual Medicaid/Medicare insurance	0.94 (0.88 – 1.00), [0.054]	n/a

Data presented as Odd ratios (OR) (95% CI) of TE ≥2, [*p* value]

## Results

Among 245 HHN practices, 136 met inclusion criteria. Over half were clinician owned and 27% were federally qualified health centers (FQHC) or FQHC look-alikes (Table 3). Approximately 40% of practices were located in a medically underserved area (MUA). Nearly 28% of practices had previously worked with the NC AHEC practice support program and 75% of practices had the same facilitator for duration of the intervention. The overall mean TE score was 1.6 (SE 0.4) and the median was 2. Among the practices that met inclusion criteria, 103/136 (76%) had contact with their facilitators during all 6 out of the 6 months included in this analyses. For those deemed ineligible that had practice level data, only 17% had this same level of contact.

By considering GLMM with a single predictor, higher leadership scores, practicing in a MUA location, and having higher percentages of uninsured patients were associated with greater odds of achieving a TE score of ≥2 at the intervention mid-point (see Table 4). Conversely, having higher percentages of dual Medicaid/Medicare insurance were associated with lower odds. When comparing independently owned practices to Federally Qualified Health Centers/health departments and separately to practices that are hospital owned or part of larger health care systems, independently owned practices were less likely to achieve a TE score of 2 or greater. Practice levels of burnout, adaptive reserve, and readiness were not associated with levels of TE.

The final GLMM model was selected based on the smallest AIC value. The following factors were associated with greater odds of a achieving a TE score of ≥2, 1) greater practice leadership and 2) practice location in a MUA. No practice facilitator characteristics were significantly associated with the outcome. None of the models that included payer mix demonstrated statistical significance.

## Discussion

Our evaluation shares insights into specific practice characteristics associated with greater odds of engagement of practice facilitators with their practice QI teams at the midpoint of the 12-month HHN CVD prevention trial. We are not aware of other studies that have quantitatively evaluated levels of engagement of facilitators with their teams in this manner.

Our data suggests that greater engagement with facilitators was associated with; 1) practices with leaders who support QI implementation and 2) practices located in MUA’s, with the former having a greater relative impact on this relationship.

Based upon our experience with other NC based practice support projects, we were not surprised that practices located in more remote areas, thus likely with fewer internal resources for implementing practice changes, may be more open and welcoming to facilitation services. We are not aware of any literature that has analyzed similar associations.

We were also not surprised to see the strong association of practice leadership with TE. Within the practice transformation literature, more effective leadership has been associated with greater engagement of practice teams in change activities [37]. In an editorial, Bohmer outlines key physician leadership activities critical to organizational change, including leadership responsibilities with 1) defining care goals, 2) ensuring that “clinical microsystems” can execute such goals, 3) engaging in data driven QI methods, and 4) modeling how to step beyond usual boundaries in order to drive organizations towards “relentless” improvement [38]. We believe that a key facet of strong leadership is the ability to create and support high functioning teams and in the case of leveraging facilitation resources, paving the way to have skilled facilitators become part of practice QI team structures. Other leaders, who are less effective, may be less able or inclined at to support high functioning teams.

Although not included in the final model selected based upon lower AIC, results of another model that included leadership and practice ownership type suggested that compared to independently owned practices, practices that are Federally Qualified Health Centers engaged with their facilitators more readily. This same signal was noted for health system/hospital/faculty practices compared to independently owned practices. Regarding practice ownership type, we suspect that those owned by health systems or hospitals may be more receptive to including facilitators into their teams as it may be a more familiar improvement strategy in such settings vs. in independently owned practices that may function more autonomously. Additionally, Federally Qualified Health centers must have an ongoing quality improvement/assurance system [39]. They are specifically expected to address ways to adhere to evidenced based guidelines, standards of care, and standards in the provision of health care services. As part of this they complete quarterly QI/Quality assurance assessments and must implement follow up actions as deemed necessary. Independently owned practices can certainly engage in a variety of programs, but are not systematically expected to do so like FQHC’s,

We were surprised by the lack of associations between practice level burnout, adaptive reserve, and organizational readiness with TE. We expected that practices with higher levels of burnout might view the study as an additional burden, thus would be more challenged with engagement. We were not able to find other studies where the burnout measure has been calculated at the practice level and did not have a vetted algorithm for generating practice level scores. Without work in establishing theoretical underpinnings and construct validity of the burnout measure at the practice level, it is difficult to understand if null findings are at least partly a measurement issue. The challenge with interpreting these null findings also applies to the adaptive reserve and organizational readiness outcomes. The adaptive reserve measure has not been rigorously validated as its own measure as it was a 23-item instrument that emerged from factor analyses performed on responses of 31 uniquely motivated practices in the National Demonstration Project [40] and the organizational readiness instrument has not been tested yet for predictive validity.

No practice facilitator level measures had independent effects on the TE outcome in our multivariable models. We suspect that there could be personality, communication, or other facilitator characteristics that may impact engagement, but we did not collect such data. Mold et al. in a study where 5 different facilitators guided 12 practices in implementing activities to enhance preventive services, found no effect of the individual facilitator on their study outcomes [41].

As stated, one of the most notable results of this analysis was the influence of greater leadership support for quality improvement and the odds of reaching a TE score of 2 at the study mid-point. This raises the question as to whether or not there are effective interventions that can help practice leaders increase their understanding and opinions of team-based QI prior to a study’s implementation. It may be important to research how to better engage practices where QI team engagement is sub-optimal, for instance to determine if certain types of activities are a better fit and/or if delving into actions that can provide “quick wins” can enhance engagement. There are potential policy implications to consider related to our findings. If the level of practice leadership for QI is critical to a practice team’s ability and willingness to engage with facilitators, then there may be opportunities to help leaders understand the value of QI by using different techniques based upon where leaders are along the change continuum. Several helpful strategies are included White paper “Engaging Primary Care Practices in QI” by Geonnotti et al. and include helping practices by 1) relieving “pain points”, 2) preparing for inevitable changes in health care quality reporting, 3) linking QI work with core values and larger missions of practice organizations, 4) demonstrating how QI can result in lower administrative task burden to clinicians, 5) exposing new practices to early adopters and enthusiastic QI opinion leaders, and 6) using “proxy relationships”, thus already trusted sources, to help make the case for QI work [15]. Understanding if there are specific actions that can enhance leaderships’ enthusiasm for QI may be an important topic for the facilitation research agenda.

Additionally, it is possible that facilitation services in non-MUA areas or with specific types of ownership are better served with different types of facilitation services. This fits squarely with a statement by McHugh et al. who shared their experiences with suboptimal practice engagement in the Heart Health in the Heartland study [17]. They noted that more research is needed to identify best strategies for practice engagement and to understand if in some cases, simple targeted practice facilitator support may be more useful than the comprehensive support provided in larger initiatives like Heart Health Now.

Of note we did not see an association between practice size, used as a continuous measure, and our outcome, while other studies have indicated that smaller practices may be more likely to engage with facilitators due to a lack internal staff members who can take on QI tasks [42]. Our study focused on small to medium sized practices only, thus this may be part of why we did not see associations with size and our outcomes as others have. In another study, practices with fewer than 3 providers demonstrated improvement in one of the overall clinical outcomes, again suggesting that practice size may matter [43]. McHugh et al., in their qualitative analysis of another EvidenceNOW collaborative’s experience, posits that smaller practices may be better supported through less complex interventions than what was included in the HHN study, thus a potential reason for our not seeing an effect of practice size in our analysis [17].

### Limitations

Our study’s findings must be considered in light of its limitations. First our practices were small to medium-sized practices that deliver primary care in NC and chose to participate in HHN, thus may not be representative of all primary care practices.

The KDIS measurement instrument was developed by experts in primary care quality improvement to guide and capture implementation efforts within primary care practices and has not been subjected to the rigorous validation processes. However, the KDIS is used in a multiple NC AHEC projects and analyses where we continue to understand its value as an implementation effectiveness measurement tool [24–26, 44]. The TE outcome measure and the KDIS Leadership measures were scored by each practice’s facilitator, thus there is a potential for same source bias.

Additionally, many practices were not eligible due to missing data. We suspect that practices that met our inclusion criteria, thus put efforts into filling out practice surveys and activity implementation, could have been more engaged with the study than those with missing data, which may potentially bias our results towards the null. Additionally, as staff turnover is common in practice, it is possible that different staff members provided responses to the baseline surveys vs. those who participated in team activities during the intervention phase, potentially complicating results interpretation.

## Conclusion

In our analysis, greater practice engagement with practice facilitators appears to be enhanced in practices located in MUAs and those with greater involvement of leadership in quality improvement efforts. The impact of leadership may be particularly important based upon this analysis and the years of facilitation experience in NC and beyond. How to engage with leaders to optimize the use of facilitation resources and how to enhance leadership support for QI are topics to include in the facilitation research agenda going forward**.** Additionally, the research community may benefit from reflecting on the experiences of Evidence NOW and other large-scale primary care research projects and work to devise practical measures that capture practice level constructs and commit to their testing and validation.

## Data Availability

The data set supporting this analysis is available upon request by contacting Dr. Jacqueline Halladay via email (Jacqueline_halladay@med.unc.edu).

## Abbreviations

GLMM: Generalized linear mixed-effects model
MUA: Medically Underserved Area
CVD: Cardiovascular disease
ABCS: Aspirin, Blood Pressure, Cholesterol, Smoking
AHRQ: Agency for Healthcare Research and Quality
HHN: Heart Health Now
QI: Quality Improvement
EHR: Electronic Health Record
FQHC: Federally Qualified Health Center
KDIS: Key Driver Implementation Scale
NC AHEC: North Carolina Area Health Education Center
TE: Team Engagement
PCS: Practice Characteristics Survey
PMS: Practice Member Survey
ICC: Intracluster correlation coefficient
AIC: Akaike information criterion
